# A severe cutaneous reaction caused by acetaminophen in a pediatric patient

**DOI:** 10.1186/2045-7022-4-S3-P140

**Published:** 2014-07-18

**Authors:** Jan Schroeder, Stefania Conio, G Pattarino, Antonella Citterio, Luca Balossi, Elide Pastorello, C De Giacomo

**Affiliations:** 1 Allergy and Immunology Unit, Niguarda Hospital, Italy; 2 Niguarda Hospital, Pediatric Ward, Italy; 3 Niguarda Hospital, Burn Unit, Italy

## 

A small girl of 2 years-6-months was admitted to our hospital for widespread rash on the face, trunk and limbs that had begun 24 hours previously; she also complained arthralgia of small and large joints and fever. She had received acetaminophen at home for 3 days because of febrile gastroenteritis. Family history was positive for allergy (mother had allergic rhinitis and beta-lactam drug allergy). The rash was widespread and included confluent purpuric macules on the trunk, limbs and on the face. There was no mucosal involvement and no skin detachment. Blood work up showed a slight increase of CRP (1.1 mg/dl) and ESR (34 mm/1h), as well as elevated tryptase (19.2 ng/ml -- nv 0-5) and total IgE (178 KU/I). Liver and kidney function was normal as well as electrolytes and coagulation; screening for autoimmunity (ANA, ENA, AMA, antiDNA, ASMA, ANCA, C3, C4, rheumatoid factor) and the infectious valutation (Ab anti CMV, EBV, HHV6 and 8, Mycoplasma, postnasal swab for virus) were negative. Skin biopsy was compatible with vasculitis from possible adverse drug reaction (slight oedema in the upper dermis associated with perivascular lymphohistiocytic infiltrate, without a relevant presence of eosinophils). The patient was treated with methylprednisolone with rapid alleviation of symptoms and a complete resolution within 10 days. The pediatric literature reports numerous hypersensitivity reactions with vascular involvement, caused by NSAIDs and beta-lactam agents (1), while cases regarding the assumption of acetaminophen are rare. Although adverse cutaneous reactions to acetaminophen are rare, considering the widespread use of this drug in pediatric age, we believe that the description of this case is useful inasmuch as a prompt diagnosis and an early treatment as well as drug suspension are crucial for improving the prognosis.
